# EDISON-WMW: Exact Dynamic Programing Solution of the Wilcoxon–Mann–Whitney Test

**DOI:** 10.1016/j.gpb.2015.11.004

**Published:** 2016-01-29

**Authors:** Alexander Marx, Christina Backes, Eckart Meese, Hans-Peter Lenhof, Andreas Keller

**Affiliations:** 1 Chair for Clinical Bioinformatics, Medical Faculty, Saarland University, Saarbrücken 66123, Germany; 2 Department of Human Genetics, Saarland University, University Hospital, Homburg 66421, Germany; 3 Chair for Bioinformatics, Saarland University, Saarbrücken 66123, Germany

**Keywords:** Wilcoxon–Mann–Whitney test, Wilcoxon rank-sum test, Dynamic programing, Exact permutation, Parallel optimization

## Abstract

In many research disciplines, hypothesis tests are applied to evaluate whether findings are statistically significant or could be explained by chance. The **Wilcoxon–Mann–Whitney (WMW) test** is among the most popular hypothesis tests in medicine and life science to analyze if two groups of samples are equally distributed. This nonparametric statistical homogeneity test is commonly applied in molecular diagnosis. Generally, the solution of the WMW test takes a high combinatorial effort for large sample cohorts containing a significant number of ties. Hence, *P* value is frequently approximated by a normal distribution. We developed EDISON-WMW, a new approach to calculate the **exact permutation** of the two-tailed unpaired WMW test without any corrections required and allowing for ties. The method relies on **dynamic programing** to solve the combinatorial problem of the WMW test efficiently. Beyond a straightforward implementation of the algorithm, we presented different optimization strategies and developed a parallel solution. Using our program, the exact *P* value for large cohorts containing more than 1000 samples with ties can be calculated within minutes. We demonstrate the performance of this novel approach on randomly-generated data, benchmark it against 13 other commonly-applied approaches and moreover evaluate molecular biomarkers for lung carcinoma and chronic obstructive pulmonary disease (COPD). We found that approximated *P* values were generally higher than the exact solution provided by EDISON-WMW. Importantly, the algorithm can also be applied to high-throughput omics datasets, where hundreds or thousands of features are included. To provide easy access to the multi-threaded version of EDISON-WMW, a web-based solution of our algorithm is freely available at http://www.ccb.uni-saarland.de/software/wtest/.

## Introduction

Hypothesis testing is used in many research areas to evaluate if findings are statistically significant or could be explained by chance. In order to evaluate hypothesis tests, the calculation of *P* values is a common method. A well-known method to calculate *P* values is the *t*-test. In contrast to the *t*-test, which requires a normal distribution of two groups, a non-parametric test, the Wilcoxon–Mann–Whitney (WMW) test, can also be applied in many cases [1]. This test is applied to elaborate if two sample groups (random variables) have equal distributions. It is commonly applied in the life and social sciences as well as in the biomedical sciences [2]. In addition, a theoretical and empirical analysis of three families of statistical tests, parametric, non-parametric, and non-parametric tests that assume non-commensurability of the results, revealed that non-parametric tests like the WMW test are preferred over parametric tests [3].

There are two different test statistics that can be used to calculate the WMW test. The first is the Mann–Whitney *U* test, which uses an approximation for the *P* value if the sample sizes exceed the number of eight elements [4]. The second test statistics, which is the focus of our current work, is the Wilcoxon rank-sum test [5]. Wilcoxon rank-sum test is a rank-based test, where the *P* value is evaluated by calculating the rank for the two samples, which is compared to the rank of all possible permutations of the samples. Theoretically, one can calculate the exact solution also for large cohorts, but most algorithms tend to approximate the result, because the computational effort to solve the exact permutation problem, which is exponentially growing with the cohort size, is too high. Nevertheless, different algorithms have been developed to solve the test exactly. One of these algorithms is a network-based approach that is based on recursion [6]. In 1997, Cheung and Klotz [7] developed an exact solution of the problem using minimal linked lists and applied their algorithm to the data used by Mehta and his colleagues [6]. Another one is implemented in the statistical library R [8], which was compared with the method described in this study. Independent of the sample size, many approaches have problems to provide an exact solution when there are tied entries in the sample data. For small sample sizes in the range of ten, it is possible to tabulate the solutions for the exact test, whereas this is not possible anymore, if the data include duplicated values [9]. Even if an approximation is used to calculate the WMW test, one has to use correction terms for ties.

Facing these problems, we developed an algorithm named Exact Dynamic Programming Solution of the Wilcoxon rank-sum test (EDISON-WMW) to solve the exact WMW test for large cohorts up to cohort sizes of about 500 for tied and non-tied data input without requiring the usage of any corrections. EDISON-WMW is based on dynamic programing, where all permutations are calculated in a stepwise procedure. This strategy allows the usage of several optimizations to reduce the computational effort. First, we are able to use filters to discriminate permutations that could have an influence on the *P* value from permutations we can be sure that do not influence the *P* value. Second, the representation that we use to enumerate the permutations allows us an efficient storage, which also allows for a parallelization of our algorithm. Moreover, the stepwise approach enables us to efficiently calculate tied samples, leading even to a decreased runtime, depending on the number of ties. Importantly, the efficiency of our algorithm is also higher for larger *P* values and decreases with the increasing significance. This is of special importance for high-throughput studies where, *e.g.*, tens of thousands of genes are measured in an integrative manner but only a small number of them are expected to be correlated to the respective study set-up.

We evaluated the performance of EDISON-WMW not only on randomly-generated datasets but also on a biomarker case-control study. Here, the expression levels for 18 microRNAs (miRNAs) were measured for 46 chronic obstructive pulmonary disease (COPD) patients and 46 lung cancer patients.

## Results and discussion

We aimed to provide an exact solution for the two-tailed unpaired WMW test without any corrections and for tied data. To this end, we compiled the null distribution of the test statistic by exact permutation in an efficient manner. We first described the actual complexity and the performance on simulated data of EDISON-WMW. Then we compared its performance with 13 other methods in terms of *P* value calculation of WMW test and benchmarked EDISON-WMW against the dataset used previously [6], [7]. Finally, we applied EDISON-WMW to miRNA profiles generated by RT-qPCR for lung cancer and COPD patients [10].

### Performance estimation and memory usage on simulated data on different versions of EDISON-WMW

First, we evaluated the performance of the algorithm on simulated data. The respective computer was equipped with 512 GB RAM and four AMD Opteron processors, totaling 64 cores with 2.4 GHz. We compared the basic version without the optimization, the optimized version, both run on a single thread, and finally the multi-threaded optimized version. The two benchmark datasets contained two groups of samples with *m* = *n* = 50 and *m* = *n* = 100 samples, respectively. To exclude the influence of ties, the dataset did not contain duplicated values. For the cohort size of 50, the basic version took 109.6 s to calculate the exact *P* value, the optimized version 1.6 s, and the multi-threaded version 1.1 s. For the second dataset, the basic version required 1897.4 s, the optimized 42.1 s, and the multi-threaded 17.2 s. The optimized version was thus about 50 times faster as compared to the basic version, and the multi-threaded implementation was even 100 times faster.

**Figure 1:**
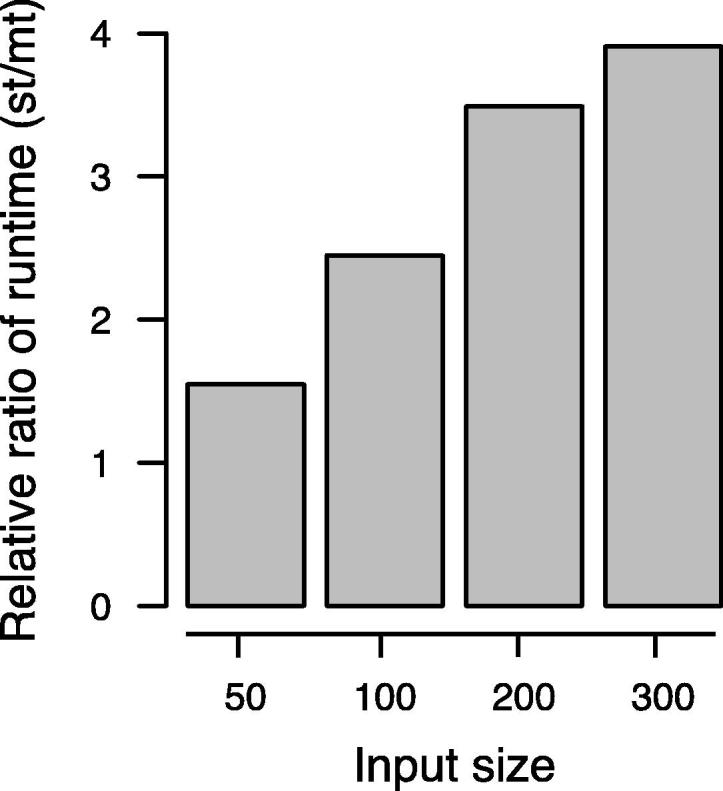
**Influence of multi threading on algorithm performance at different input size** For larger sample sizes, a fourfold increase of performance is reached in comparison to the optimized single threaded approach. The *Y* axis denotes the relative ratio of the running time of the single-threaded (st) and multi-threaded (mt) versions of EDISON-WMW.

The difference between the optimized single-threaded and multi-threaded versions became even more evident using larger cohorts, as shown in Figure 1. Compared to the optimized single threaded version, for 50 samples per cohort, the speed was increased 1.55-fold using multithreaded version. The improvement in performance is increased with the increase in cohort size. For a cohort with 300 samples, the speed is increased up to 3.91-fold, with less than 3 GB RAM. In particular, our algorithm performed well for tied data. For example, by increasing the number of ties, the computation time for cohorts of size 50 was reduced to 0.27 s and for cohorts of size 100 in 2.87 s. For *m* = *n* = 500 samples with ties, the exact results were obtained in around 12 min, demonstrating that even for such large cohorts, exact *P* values can be obtained in reasonable time.

We further test our algorithm on categorical data with large sample sizes for two datasets. The datasets included four categories mapped to integer values with the sample sizes of 1000 and 2000, respectively. Results for 1000 samples per group could be obtained in less than 10 min with about 9 GB RAM. Due to the exponential runtime and the very large sample size, the test for 2000 took about 96 min with less than 110 GB RAM to obtain the result.

### Number of duplicates in the hash table

**Figure 2:**
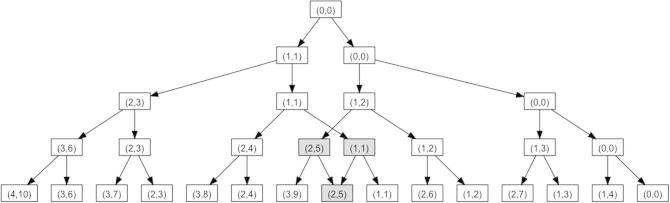
**Complete binary tree up to the fourth iteration** The first duplicated entry occurs in layer 4, which is highlighted in gray. Same results were also obtained for 2 entries in layer 3.

**Figure 3:**
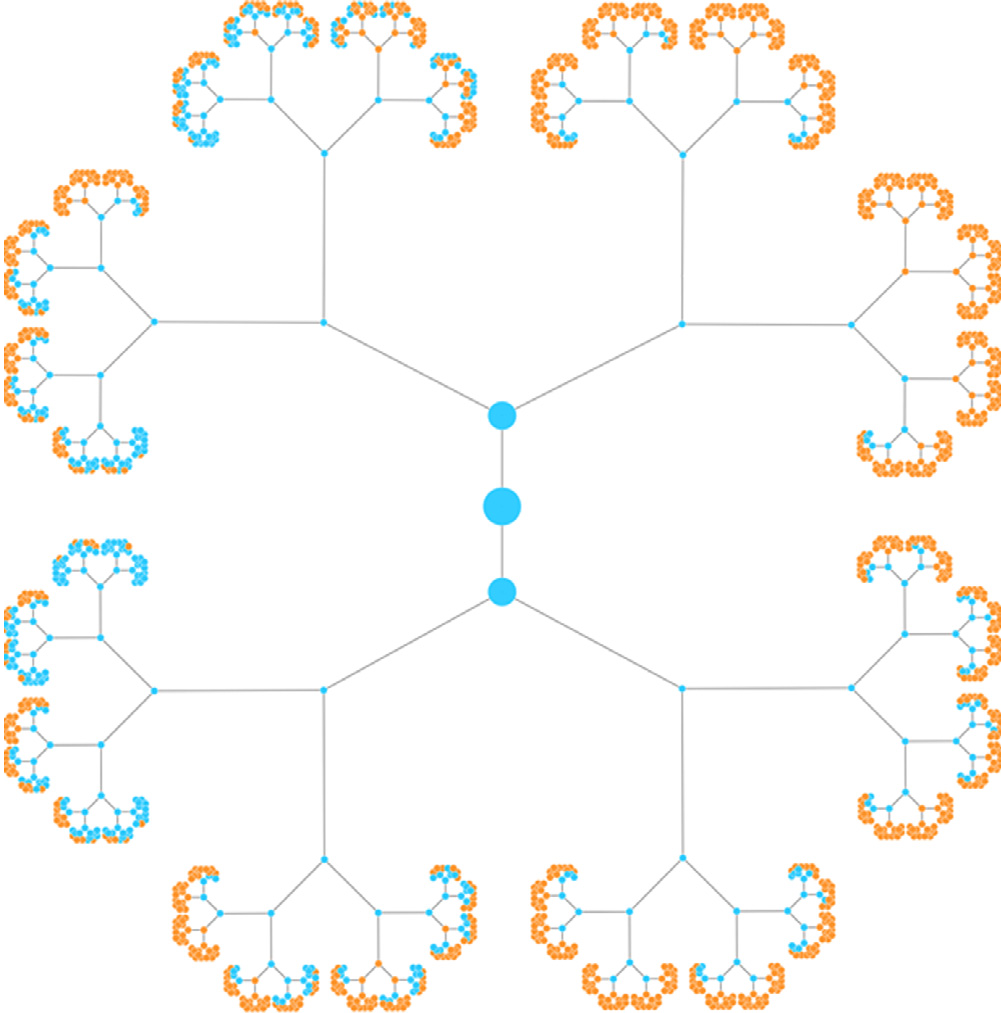
**Tree for the first 7 iterations** The root node is presented in the middle, and a binary tree is constructed from this root node. The blue nodes represent nodes that have to be considered while the orange nodes represent duplicates. The first duplicate is discovered in the fourth iteration (upper right part of the image).

A key characteristic of our algorithm is that the tree, which is built up layer by layer, is actually not binary. We would have, after *i* iterations, 2*^i^* leaves and a total of 2*^i^*^+1^ − 1 nodes, if building up a binary tree. For example, there would be 2047 nodes after 10 iterations and more than 2 million nodes after 20 iterations. As shown in Figure 2, starting from the fourth layer on, we however observed replicated entries in the tree. We thus calculated the number of replicates. There is one replicate at layer four (6.6%) and at layer 10, we find 72.59% replicated values, thus not 2047 but just 561 entries have to be stored in the hash table. After 20 steps, 99.64% replicates are detected. Hence, 7550 nodes instead of over 2 million nodes in the tree are considered. After 28 iterations, 99.999% of all entries in the tree were duplicates. The rapidly increasing amount of duplicates is presented in Figure 3. Here, the first 8 layers of the tree are provided, the root node is shown in the middle. From layer to layer, the duplicated entries that are highlighted in orange increase substantially. The more such replicates we obtain, the higher the performance of our algorithm actually is.

### Dependence of runtime from *P* value

**Figure 4:**
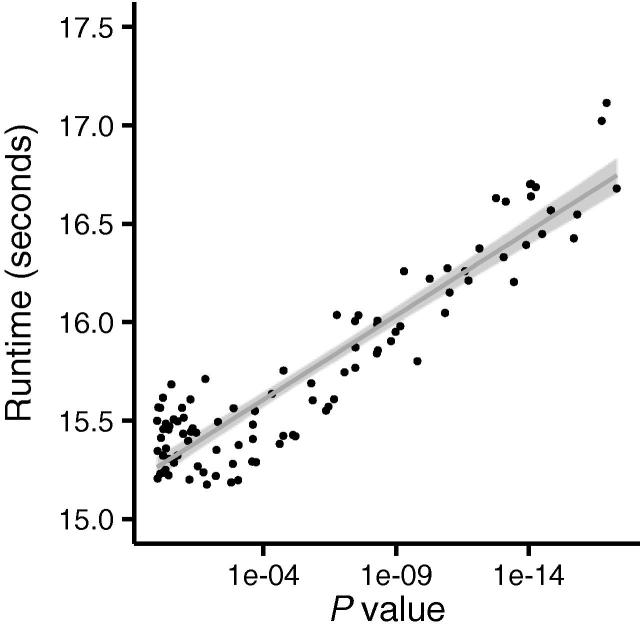
**Correlation between runtime and significance value** Running time of test runs was plotted based on the resulting *P* value. The *X* axis presents the negative decimal logarithm of the *P* value. Here, higher values correspond to lower significance values and thus highly significant results. Runtime is shown in seconds on the *Y* axis. *m* = *n* = 50 for the cohort size. The black line was generated using linear regression and the gray area around the line denotes the standard error.

As shown above, there is a strong dependence of the runtime of our algorithm on the number of ties. We then evaluated whether there is a dependence of runtime on the significance value. We generated random datasets with R across a wide range of *P* values, from really low (10^−10^) to *P* values close to the maximum of one (10^−1^). The cohort size has been fixed to *n* = *m* = 100 samples and in order to exclude the influence of ties, no ties were allowed. As the scatter plot in Figure 4 details, we actually discover a higher runtime for very significant results, which is expected since fewer elements can be pruned by our algorithm in this case. This behavior is of interest for high-throughput omics datasets, where many thousands of features can be tested in an integrative manner. In respective studies, the portion of non-significant features (requiring less runtime) is usually substantially higher than that of significant (*P* < 0.05) or very significant (*P* ≪ 0.05) findings.

### Comparison to other statistics packages

As described previously, different versions of EDISON-WMW have been developed to solve the WMW test exactly. Prominent examples are a network-based approach [6] and a linked list-based C implementation [7]. Both evaluated a dataset from a double-blind randomized study on a new agent in patients with rheumatoid arthritis. The cohort sizes were *m* = 107 and *n* = 122. Our algorithm calculated the result far below one second. While a direct comparison of the runtime was not possible, the speed-up from the naïve implementation, as shown above in the section about the performance of EDISON-WMW, is a good indicator of the performance. Consequently, Metha and his colleagues obtained a 19-fold improved runtime, which is well in line with, although not as good as, the improvements observed for EDISON-WMW (basic version and optimized version, notably without parallelization).

Bergmann et al. [9] compared 11 statistical packages for calculating WMW test *P* values with a cohort of *m* = *n* = 12 rats including ties. Of the 11 packages, four were able to calculate the exact permutation, however, just for limited cohort sizes. The actual *P* value for the scenario given in Bergmann et al. was 0.0373, while the programs tested delivered *P* values between 0.0147 and 0.089. Only a small fraction of the tested programs, including SPSS and ArcusQuickstat, delivered the exact result. Therefore, these packages are limited, considering the cohort size.

In our current study, besides these 11 programs, we also tested the dataset with “R”. “R” calculated *P* values of 0.01647 and 0.01473, with and without continuity correction, respectively. In both cases, “R” draws a warning that for tied data no exact *P* values can be calculated. Additionally, we also checked various web servers that are freely available to calculate WMW test *P* values. For instance, using the service of Phonetic Sciences in Amsterdam (http://www.fon.hum.uva.nl/Service/Statistics/Wilcoxon_Test.html), we got the result *P* ⩽ 0.08853. Using the same data as input, all three versions of EDISON-WMW delivered the exact result according to Bergmann et al. in far below one second computing time.

**Figure 5:**
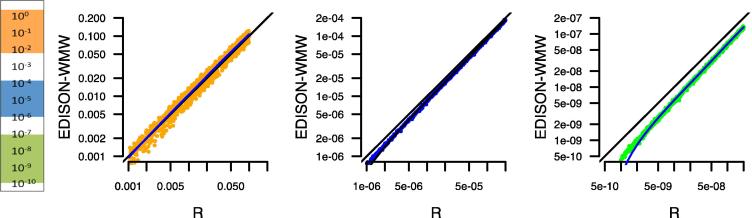
**Benchmarking of *P* value between R and EDISON-WMW** Performance of R and EDISON-WMW was compared for calculating *P* values in the range of 10^−10^–1. Three ranges are highlighted, not significant or moderate significant (down to 0.001) in orange, more significant (down to 10^−6^) in blue, and very significant (down to 10^−10^) in green. For low *P* values, “R” seems to under-estimate the significance by overestimating the *P* values.

Since “R” is one of the most commonly applied tools for statistical calculations, we performed an additional comparison in order to check how far R deviates from the exact solution. In Figure 5, we evaluated *P* values between 1 and 10^−10^ for randomly-generated data. While in the moderate *P* value range (10^0^–10^−2^; orange), just minor deviations from the exact solution were discovered, we observed, especially in low *P* value ranges (10^−7^–10^−10^; green), a bias of R toward less significant results, which is already evident for the intermediate *P* value ranges (10^−4^–10^−6^; blue). This may become problematic especially in high-throughput studies, since the required adjustment for multiple testing may worsen the described effect.

### Evaluation of lung cancer and COPD miRNA patterns

So far we presented evidence for the correctness and efficiency of our algorithm. To demonstrate the performance on real molecular diagnostic data, we evaluated a set of *m* = *n* = 46 samples from lung cancer and COPD patients. These samples have been screened using RT-qPCR for 18 miRNAs related to lung cancer and COPD. The *P* values were calculated using EDISON-WMW and “R” with the correction for continuity enabled. Finally, the *P* values were adjusted for multiple testing using the Benjamini Hochberg approach [11]. The raw *P* values and corrected significance values are shown in Table 1. In most cases, R and EDISON-WMW showed a very good concordance. Both “R” and EDISON-WMW reported significant results according to raw *P* values (*P* < 0.05, highlighted in gray) for 6 miRNAs. Following adjustment for multiple testing, 5 miRNAs remain significant using EDISON-WMW. Moreover, in all 5 cases the exact *P* values obtained using EDISON-WMW was lower than those calculated by “R”. For has-miR-93^∗^, the exact *P* value calculated by EDISON-WMW remains significant (0.048) but not any more using “R” (0.05). In sum, these data demonstrate our exact implementation of the WMW test can be applied to evaluate molecular biomarker profiles efficiently.

## Conclusion

Here we present an algorithm for the exact solution to the two-tailed unpaired Wilcoxon–Mann–Whitney test, even in the presence of ties. Our algorithm performs especially well for data that contain many ties and show higher significance values. These data are frequently generated by high-throughput molecular approaches, such as in genomics and transcriptomics. We benchmarked EDISON-WMW against other tools for calculating WMW test *P* values using simulated data and real miRNA profile data. We show that our algorithm can be applied even if 1000 samples are included in the study. To facilitate access to the EDISON-WMW, we implemented an easy to handle web-based solution, on which users are able to fill in their data manually or upload their data in CSV-format for *P* value calculation.

## Materials and methods

### Calculation of the WMW test

The WMW test can be calculated with the Mann–Whitney *U* test and with the Wilcoxon rank-sum test. We implemented the Wilcoxon rank-sum test to provide an exact solution.

We sort all elements of the two given random samples *X* and *Y* of sizes *m* and *n* in an increasing order and store them in a list *L* of length *m* + *n*. If the list does not contain elements with the same value (ties), the rank of each element is equal to its position in *L* starting with rank one. Tied elements receive each the arithmetical mean of their positions as ranks. The rank of a set S⊆L is defined as the running sum $r(S)=\sum_{s\in S}r_{s}$ of the ranks of its elements [5]. In order to calculate the *P* value, we compare the rank *r*(*X*) of *X* to the ranks of all group label permutations of *L*, *i.e.*, all subsets of *L* of size *m*, and do the same for *Y*. The two-sided *P* value for two samples *X* and *Y* with |*X*| = *m* and |*Y*| = *n* is then defined as

(1) $$P=2\cdot \min\left(\frac{\pi_{r(X)}}{\left(\begin{array}{c} m+n\\ m \end{array}\right)}\text{,}\frac{\pi_{r(Y)}}{\left(\begin{array}{c} m+n\\ m \end{array}\right)}\right)$$

where *π_r_*_(_*_X_*_)_ is the number of all subsets of size *m* that have a lower or equal rank than *r*(*X*) and, equivalently, *π_r_*_(_*_y_*_)_ is the number of all subsets of size *n* that have a lower or equal rank than *r*(*Y*).

### Dynamic programing

The number of permutations (subsets of size *m* and *n*, respectively) is increasing exponentially with the increasing cohort size. Using this approach, we show how the number of ‘considered’ subsets can be effectively reduced by the usage of filters and how these subsets and their ranks can be stored efficiently. Moreover, we demonstrate how the calculation of tied samples can be done efficiently and how multi-threading support can be added to further speed up the computation.

Using a dynamic programing approach, we compute *π_r_*_(_*_X_*_)_, the number of subsets of list *L* of size *m* with ranks not exceeding the calculated rank *r*(*X*) of *X* (and equivalently *π_r_*_(_*_Y_*_)_). To this end, we iterate backward over all elements of *L* starting with the last element at position *n* + *m* with rank *r_n+m_*. In order to solve the aforementioned counting problem, we have to compute the number of subsets of size *m*

(2) $$\pi_{r(X)}:=\pi_{r(X)}(m\text{,}n+m)$$

whose ranks do not exceed the calculated rank *π_r_*_(_*_X_*_)_, *i.e.*, all subsets of size *m* whose running sums up to the last position *n* + *m* are smaller or equal to *π_r_*_(_*_X_*_)_. In order to derive a recursion formula, we have introduced two further parameters: the first parameter *m* describes the size of the considered subsets; the second parameter *n* + *m* defines a position in the list. The following recursion holds for a given positive threshold *S*, a position *i*, and a subset size of *k*

(3) $$\pi_{S}(k\text{,}i)=\pi_{S}(k\text{,}i-1)+\pi_{S-r_{i}}(k-1\text{,}i-1)$$

Here, *π_S_*(*k*, *i* − 1) counts the subsets excluding element *i*, whereas $\pi_{S-r_{i}}(k-1\text{,}i-1)$ counts the subsets containing element *i*. In the latter case, the rank *r_i_* will be added to the running sum and, hence, the threshold has to be reduced to *S* − *r_i_*. Furthermore, the number of elements that still have to be selected during the remaining recursive steps has to be reduced to *k* − 1.

Given the sorted list *L* with the ranks of all elements, the recursive calculation of the number of valid subsets can be represented by a binary tree that can be constructed in two directions, either starting from the beginning or from the end of list *L*. In the implementation described below, we build up the tree starting from the first element. Using dynamic programing, we count the number of valid subsets iteratively based on this tree. The tree is calculated stepwise starting with the root node and adding in each step another layer. Since layer *i* depends only on the previous layer *i* − 1, only the current and the previous layers have to be stored. Another important aspect of the algorithm is an efficient storing method for the calculated recursion elements. We use a hash map, which takes as key a pair (*k*,*r*) consisting of the subset size *k* and the rank-sum *r* of this subset. The number of subsets of size *k* and rank-sum *r* is stored.

Figure 2 shows an example of a sample tree of height four, where the input does not include ties. To get the exact *P* value, we have to perform *m* + *n* steps, *i.e.*, layer computations. Before step one, we initialize the tree with the root element (0,0), which obtains the value ‘1’. In every step, we create for each entry in the old hash map two entries in a new hash map: the entry itself and another entry that adds the information of the actual element *i*, namely the number of added elements (‘1’) and the rank *r_i_* of element *i*, to the key of the original entry. This means for the second element the key will by increased by (1,*r_i_*). In Figure 2, the respective keys of the hash map are shown, where the first four keys (the left arm of the tree) contain the count of elements, 1–4 (left part of the keys) and the sum of 1 to the number of contained elements (right part of the keys). For example the key (3, 6) represents a set containing 3 elements and the sum of them is 6 (sum of 1–3). With the exception of the element highlighted in gray in the last line, the values of all elements are equal to one. In row three, the elements (2,5) and (1,1) both have the child (2,5) and therefore the value of this element is two. For larger tree sizes, the case that two parent nodes share the same child node happens very frequently, which leads to a substantially-decreased memory usage and running time. More details on running time and memory usage were provided in the Results and discussion section.

### Extension of the basic algorithm

To accelerate the computation, the basic version of the algorithm presented above has been extended in several directions. In order to decrease the memory usage and the running time of the algorithm, four filters have been introduced. Since only the valid subsets that belong to *π_r_*_(_*_X_*_)_ and *π_r_*_(_*_Y_*_)_ have an influence on the *P* value, we can remove non-valid elements from the hash map immediately. Consequently, for two samples *X* and *Y* with |*X*| = *m* and |*Y*| = *n*, where *i* is the actual step and *r_next_* is the sum of the ranks of the next *m* − *k* elements, we can remove an entry (*k*, *ν*) if one of the following rules is fulfilled:

(4) $$\begin{array}{c} 1.\;k>m\\ 2.\;k+((m+n)-i)<m\\ 3.\;\nu +r_{\mathrm{next}}>r(X)\text{,}\\ 4.\;k=m\wedge v\geq r(X) \end{array}$$

Analogous rules can be applied for the second sample group *Y*.

Next, a feature that optimizes the algorithm for tied samples has been developed. If there are tied elements in the samples, we have to add the same sub-tree in several steps of the algorithm. To accelerate the computation we pre-compute the complete sub-tree for such tied sequences and then merge it with the rest of the tree. More precisely, assume that we have *k* elements with the same rank *r*, then the subtree has the form of Pascal’s triangle. Consequently, it has only *k* + 1 leaves, where the keys can be written as (*i,r* · *i*), *i* = 0, …, *k*. Based on the structure of such a subtree, the values are given by the following formula.

(5) $${\text{value}}_{\left(i\text{,}r\cdot i\right)}=\left\{\begin{array}{cc} 1\text{,} & \text{if}\;i=1\\ \left(\begin{array}{c} k\\ i \end{array}\right)\text{,} & \text{otherwise} \end{array}\right)$$

This feature is especially important for datasets where only few different numbers occur and therefore the number of ties is high.

### Implementation details

In order to get the best possible performance, we implemented the algorithm in C++. To guarantee a correct solution also for large cohorts, the class library for numbers (CLN) library [12] was used to store the values, which in such cases would exceed normal data types. Furthermore, to optimize the running time we used multi-threading for tree construction. Since the standard HashMap supported by C++, which we used at the beginning to represent our mapping, does not allow a concurrent iteration, we used the hash map from the Intel® threading building blocks (TBB) library [13] to access the map concurrently. Additionally, the *parallel_for* method of TBB was used for the parallelization.

### Details of the datasets

The simulated data have been generated using R [8]. We used normally-distributed data, whereas the standard deviation of each sample pair (*X*, *Y*) was equal and the mean sample *Y* was shifted to generate another sample group. In the case of Figure 5, we used group *X* as constant and iteratively increased the mean of group *Y* by a small amount. The lung cancer and COPD data have been described previously [10].

## Authors’ contributions

AM implemented the main part of the algorithm and the web interface, CB supported the development of the algorithm, EM contributed in data analysis and writing the manuscript, HPL and AK developed the algorithm and wrote the manuscript. All authors read and approved the final manuscript.

## Competing interests

The authors declare that they have no competing interests.

## Figures and Tables

**Table 1:** *P* values for miRNA profiles between COPD and lung cancer patients

*Note: P* values <0.05 are highlighted in gray. Benjamini Hochberg was used for adjustment.
